# Global realized niche divergence in the African clawed frog *Xenopus laevis*

**DOI:** 10.1002/ece3.3010

**Published:** 2017-05-10

**Authors:** Dennis Rödder, Flora Ihlow, Julien Courant, Jean Secondi, Anthony Herrel, Rui Rebelo, G. J. Measey, Francesco Lillo, F. A. De Villiers, Charlotte De Busschere, Thierry Backeljau

**Affiliations:** ^1^ Herpetology SectionZoologisches Forschungsmuseum Alexander Koenig (ZFMK)BonnGermany; ^2^ UMR7179 CNRS/MNHNParisFrance; ^3^ UMR5023 Ecologie des Hydrosystèmes Naturels et AnthropisésENTPECNRSUniversité de LyonUniversité Lyon 1VilleurbanneFrance; ^4^ UMR CNRS 6554 LETG‐LEESAUniversity of AngersAngersFrance; ^5^ Centre for Ecology, Evolution and Environmental ChangesFaculdade de Ciências da Universidade de LisboaLisboaPortugal; ^6^ Centre for Invasion BiologyDepartment of Botany & ZoologyStellenbosch UniversityStellenboschSouth Africa; ^7^ Via Leonardo da Vinci 621020 Taino (VA)Italy; ^8^ Royal Belgian Institute of Natural SciencesBrusselsBelgium; ^9^ Evolutionary Ecology GroupUniversity of AntwerpAntwerpBelgium

**Keywords:** fundamental niche, invasive potential, invasive species, *n*‐dimensional hypervolume, niche evolution, niche shift

## Abstract

Although of crucial importance for invasion biology and impact assessments of climate change, it remains widely unknown how species cope with and adapt to environmental conditions beyond their currently realized climatic niches (i.e., those climatic conditions existing populations are exposed to). The African clawed frog *Xenopus laevis*, native to southern Africa, has established numerous invasive populations on multiple continents making it a pertinent model organism to study environmental niche dynamics. In this study, we assess whether the realized niches of the invasive populations in Europe, South, and North America represent subsets of the species’ realized niche in its native distributional range or if niche shifts are traceable. If shifts are traceable, we ask whether the realized niches of invasive populations still contain signatures of the niche of source populations what could indicate local adaptations. Univariate comparisons among bioclimatic conditions at native and invaded ranges revealed the invasive populations to be nested within the variable range of the native population. However, at the same time, invasive populations are well differentiated in multidimensional niche space as quantified via *n*‐dimensional hypervolumes. The most deviant invasive population are those from Europe. Our results suggest varying degrees of realized niche shifts, which are mainly driven by temperature related variables. The crosswise projection of the hypervolumes that were trained in invaded ranges revealed the south‐western Cape region as likely area of origin for all invasive populations, which is largely congruent with DNA sequence data and suggests a gradual exploration of novel climate space in invasive populations.

## 1 Introduction

With today's computational power and the increasing availability of both comprehensive distribution data sets and high‐resolution environmental data, the study of species niche dynamics has become a growing field of research (e.g., Crisp & Cook, 2012; Pearman, Guisan, Broennimann, & Randin, 2008; Peterson, 2011). This development was induced by the urgent need to understand and forecast effects of climate change and biological invasions on biodiversity (Sillero, Reis, Vieira, Vieira, & Morales‐Hojas, 2014; Thomas et al., 2004). These forecasts are based on the assumption that species retain inherited ecological traits and environmental niches over time and space (niche conservatism) (see Wiens & Graham, 2005 and references therein). However, information on the degree and/or how fast species can adapt to environmental conditions beyond their realized niches (i.e., those climatic conditions existing populations are exposed to) is currently limited. Therefore, assessments of niche conservatism need to cover a broad range of temporal scales to detect under which circumstances niche shifts may occur and in which time frame they are possible (Peterson, 2011). If invasive species exhibit niche conservatism, predictive niche models can be used to determine where a certain species can become established (Wiens & Graham, 2005). Thus, biogeographic patterns of invasion success offer unique opportunities to study the (pre‐)adaptive potential of the Grinnellian niches (i.e., environmental, nonconsumable niche axes operating on a macroecological scale) of species as well as their spatiotemporal dynamics (Guisan, Petitpierre, Broennimann, Daehler, & Kueffer, 2014; Pearman et al., 2008; Peterson, 2011).

Recent evidence proposes that climatic niches may not be as conserved as previously assumed and shifts of realized niches have been suggested for a number of invasive taxa including plants (e.g., Broennimann et al., 2007; Mitchell et al., 2006), insects (Fitzpatrick, Weltzin, Sanders, & Dunn, 2007; Medley, 2010), reptiles (Rödder & Lötters, 2009; Rödder, Schmidtlein, Veith, & Lötters, 2009), and amphibians (Tingley, Vallinoto, Sequeira, & Kearney, 2014). Although conflicting evidence exists for several taxonomic groups (Alexander & Edwards, 2010; Petitpierre et al., 2012; Strubbe, Broennimann, Chiron, & Matthysen, 2013), these shifts are assumed to correspond to the release from dispersal barriers or biotic constraints rather than representing novel physiological adaptations (fundamental niche shifts; Rödder et al., 2009; Tingley et al., 2014). A recent study suggests that environmental niches might be conserved within phylogenetic lineages (Schulte et al., 2012). However, few studies exploring the genetic foundation of the rate of adaptation and the role of intraspecific spatial variation in environmental niche structure exist. Furthermore, those which are available cover only few taxonomic groups (e.g., Krehenwinkel, Rödder, & Tautz, 2015; Lavergne & Molofsky, 2007; Schulte et al., 2012; Sillero et al., 2014; Vandepitte et al., 2014). Shifts in fundamental niches are only traceable in experimental setups and are difficult to disentangle using correlative models (Peterson, Papes, & Soberón, 2015). Therefore, most studies exclusively focus on shifts or stasis in realized niches.

Anthropogenic introduction pathways facilitate the spread of invasive species across biogeographical barriers and well beyond the species natural dispersal capacities (Wilson et al. 2009). This increased potential for large distance dispersal makes novel parts of the global environmental space colonizable, which may not be available in the native range. As unanticipated experiments, biological invasions represent model systems to study niche conservatism and dynamics (Jimenez‐Valverde et al., 2011; Tingley et al., 2014). From a management perspective, the identification of variables restricting an invasive species’ range is pivotal for successful management of established populations and to prevent further spread (e.g., Jimenez‐Valverde et al., 2011). The comparison of realized niche spaces of native and invasive populations facilitates the identification of range limiting constraints of environmental variables that often correspond to physiological limits (Rödder, Solé, & Böhme, 2008; Rödder et al., 2009).

Species distribution modeling (SDM) approaches have become promising tools for both basic and applied research (Kozak, Graham, & Wiens, 2008; Peterson et al., 2015) and have been frequently applied to predict the establishment success prior to issuing import permits for species of biosecurity concern (Bomford, Barry, & Lawrence, 2010; Kopecký, Patoka, & Kalous, 2016; Kumschick & Richardson, 2013). By comparing the environmental conditions of a species’ native range with the conditions at the area of planned introduction, climate matching quantifies the risk of establishment, for example, for amphibian species in the European Union (Kopecký et al., 2016), plant taxa in Australia (Kumschick & Richardson, 2013), or freshwater fishes worldwide (Bomford et al., 2010). As such climate matching approaches assume climate niches to be conserved during the invasion process, they exclude the possibility that the native range does not correspond to the entire set of conditions a species can live in with its standing genetic background or that evolutionary processes expand the fundamental niche. The niche conservatism assumption has been frequently challenged in the past (Broennimann et al., 2007; Early & Sax, 2014; Rödder & Lötters, 2010; Urban, Phillips, Skelly, & Shine, 2008) partly because it neglects nonclimatic range limitations that also shape species spatial distributions. In addition, these biotic and abiotic factors might differ between the native and the invaded range (Colwell & Rangel, 2009; Early & Sax, 2014). While such limitations are undeniably extremely important for some species (e.g., regarding species interactions), these effects have been demonstrated to be highly scale dependent, for example, in the case of facultative predator–prey systems and rarely affect distributions at large extent and resolution if they depend on abundances (Soberón & Nakamura, 2009). Predator and prey may coexist if the abundance of the predator is below the carrying capacity of the local habitat. On the other hand, biotic interactions may be limiting irrespective of scale obligatory systems, for example, in specific butterflies where larvae require a specific host plant. Furthermore, widespread, ecologically dominant species may be less affected by fine scale bionomic effects (Early & Sax, 2014).

The African clawed frog *Xenopus laevis* (Daudin, 1802) is a predominantly aquatic amphibian species native to the Mediterranean climate zone in southern Africa (Measey et al., 2012). The species was widely used for human pregnancy testing and distributed pan‐globally as a laboratory species for scientific research (Measey et al., 2012; van Sittert & Measey, 2016) where escapees and voluntarily released individuals established numerous invasive populations outside its native range (Measey et al., 2012). As *X. laevis* is abundant and widely distributed in its original range, has a high genetic variability, rapid growth, early sexual maturity, a broad diet, performs overland dispersal, is tolerant to various environmental conditions, and readily accepts heavily modified anthropogenic habitats, the species possesses great invasion potential (Measey et al., 2012, 2016). Among invasive amphibians, *X. laevis* has one of the highest recorded impacts on native fauna (Measey et al., 2016). These comprise competition for resources, direct predation of native amphibians (Amaral & Rebelo, 2012; McCoid & Fritts, 1980; Measey et al., 2015), a negative impact to the reproduction of native amphibians (Lillo et al., 2011) and has been shown to be an asymptomatic carrier of the chytridiomycosis fungus *Batrachochytrium dendrobatidis* (Fisher & Garner, 2007).

Being a model organism in a broad range of scientific disciplines, a variety of information on environmental constraints on *X. laevis* is available, including data on climatic factors that determine diurnal and annual activity patterns, reproduction and thermal tolerances (Balinsky, 1969; Casterlin & Reynolds, 1980; Eggert & Fouquet, 2006; McCoid & Fritts, 1989; Miller, 1982; Nelson, Mild, & Lovtrup, 1982; Wilson, James, & Johnston, 2000; Measey, 2016). Hence, comparisons of experimentally quantified properties of its fundamental niche with its realized niches are possible by comparing environmental conditions as observed at species occurrences with physiological and behavioral information obtained from the literature. This information may provide important insights into niche dynamics and the predictive ability—or shortcomings—of correlative SDMs.

Most specimens of *X. laevis* were exported through a single organization and represent a genetic lineage originating from the south‐western Cape region of South Africa (De Busschere et al., 2016; Lillo, Dufresnes, Faraone, Lo Valvo, & Stock, 2013; Lobos, Mendez, Cattan, & Jaksic, 2014; van Sittert & Measey, 2016). However, other lineages were detected in France suggesting that this invasive population is composed of animals from multiple source populations (De Busschere et al., 2016). Although *X. laevis* predominantly colonizes areas with equivalent climatic conditions, populations have also been established in temperate regions characterized by an oceanic climate (Fouquet & Measey, 2006; Measey & Tinsley, 1998; Rubel & Kottek, 2010). These conditions likely represent the edge of the species’ physiological capacity, and possibly extend beyond environmental conditions within the native range.

Being invasive on multiple continents, including Europe, South, and North America, *X. laevis* represents a pertinent model organism to study realized niche dynamics during invasion processes. In this study, we assess whether the realized niches of invasive populations represent subsets of the realized niche in the species’ native distributional range or if niche shifts are detectable. If so, human‐mediated dispersal may have facilitated the exploration of fundamental niche space beyond the species’ realized niche in its native range. Alternatively, niche shifts could indicate novel physiological adaptations. We expect potential niche shifts to be restricted to a subset of environmental variables as selective pressures may operate differently depending on the climatic setup of the area of introduction. This expectation appears to be reasonable given that environmental variables may interact very differently with the species’ physiology: There may be hard limits such as critical thermal minimum or maximum being lethal or variables representing rather soft limits, for example, affecting the species behavior such as triggering reproduction. From a geographic point of view, the combination of environmental conditions may strongly vary across space creating potentially very different selective landscapes. Therefore, we ask whether the realized niches of the invasive populations still harbor information of the niche of the ancestor, that is, if it is possible to reconstruct the origin of the source populations in the native range.

## 2 Materials and Methods

### 2.1 Data acquisition and preparation for environmental niche analyses

As environmental layers, we obtained remotely sensed bioclimatic variables (Beaumont, Hughes, & Poulsen, 2005; Busby, 1991) with a spatial resolution of 3 arc min (Deblauwe et al., 2016), representing minima, maxima, and average values of monthly, quarterly, and annual ambient surface temperature as well as precipitation (Table 1). Climate data represent averages of the periods 1981–2013 and 2001–2013 derived from MODIS LST and CHIRPS v.2.0 data bases. As the subsequent analyses require an orthogonal environmental space, we performed a principal component analysis (PCA) on the original bioclimatic predictors in Cran R (R Core Team, 2015) and retained principal components (PCs) with eigenvalues >1 (Table 1). A total of 925 occurrence records of *X. laevis* containing 826 records from the species’ native range in southern Africa, 38 records from Europe, 24 from North America, and 37 from South America were obtained from Ihlow et al. (2016). We follow the taxonomic interpretation by Furman et al. (2015) confining the native range of *X. laevis* sensu stricto to southern Africa (South Africa, Lesotho, Swaziland, Namibia, Zimbabwe, and parts of Botswana, Mozambique and Malawi).

**Table 1:** Summary of the principal component analysis results including Pearson's correlation coefficients, Eigenvalues, and explained total variance. Lowest and highest values per column displayed in bold

Variable	PC 1	PC 2	PC 3	PC 4
Annual mean *T*	0.527	0.508	−0.600	−0.230
Mean annual range (mean of monthly (*T* max—*T* min))	0.726	0.402	**0.460**	0.096
Isothermality (BIO 2/BIO 7) × 100	−0.333	0.680	−0.064	−0.075
T seasonality (SD × 100)	0.833	−0.358	0.341	0.087
Max T of warmest month	**0.960**	0.137	−0.054	−0.026
Min T of coldest month	−0.254	0.010	−**0.927**	−0.228
T annual range (BIO 5—BIO 6)	0.859	0.098	0.458	0.103
Mean T of wettest quarter	0.225	0.637	0.196	−**0.623**
Mean T of driest quarter	0.242	−0.232	−0.744	0.444
Mean T of warmest quarter	0.899	0.054	−0.305	−0.123
Mean T of coldest quarter	−0.114	0.575	−0.771	−0.203
Annual precipitation	−**0.908**	0.236	0.157	0.070
Precipitation of wettest month	−0.748	0.476	0.111	0.313
Precipitation of driest month	−0.522	−**0.717**	0.028	−0.351
Precipitation seasonality (CV)	0.118	**0.710**	−0.069	**0.616**
Precipitation of wettest quarter	−0.750	0.492	0.127	0.299
Precipitation of driest quarter	−0.539	−0.698	0.059	−0.364
Precipitation of warmest quarter	−0.678	0.515	0.378	−0.199
Precipitation of coldest quarter	−0.200	−0.669	−0.274	0.559
Eigenvalues	7.289	4.573	3.324	1.960
Explained Variance	38.364	24.066	17.493	10.316

### 2.2 Quantification of potential niche shifts

In order to quantify and assess potential niche shifts in the African clawed frog, we performed both univariate analyses using density profiles and multivariate hypervolume analyses (Blonder, 2016; Blonder, Lamanna, Violle, & Enquist, 2014) for the species’ native distributional range in southern Africa and all known invasive populations in Europe, South, and North America. For univariate comparisons, we computed density profiles using the relevant functions of the *sm* package for Cran R (Bowman & Azzalini, 2014). The multivariate hypervolume analysis is designed to capture the environmental niche of the target species (or its populations) following Hutchinson's original idea of a Grinnellian niche space as an *n*‐dimensional hypervolume (Blonder et al., 2014; Hutchinson, 1957). By computing the geometry and topology of the native and all invasive populations, hypervolumes can be quantified and compared in terms of shape, total volumes, niche positions, intersections, and unique parts (Blonder et al., 2014; Guisan et al., 2014). As environmental background an area defined by a circular buffer of 200 km surrounding all records from the species’ native range was used in order to capture the available climate space and hence the potential for pre‐adaptation.

The distribution of species occurrence records in orthogonal niche space is generalized based on multivariate kernel density estimations across principal components to remove effects of spatial autocorrelation and different availabilities of specific environmental combinations in geographic space (for details, see Blonder et al., 2014; Blonder, 2015, 2016). Based on the kernel density estimations a new set of random records with a homogenous density across the environmental space captured by the original occurrence records was created. The total hypervolume is derived from this set of random records.

This procedure allows the determination of the total niche volume of a species regardless of the geometry of the niche space (or within its respective native or invasive range) and is comparatively insensitive to low sample size. This niche volume can be projected back into geographic space. The resulting maps indicate areas exhibiting environmental conditions which are part of the species’ niche volume (realized niche/realized distribution). The overlap between hypervolumes was determined using the Soerensen index [i.e., for hypervolumes A and B: S = 2*|A int B|/(|A| + |B|)]. We used the *hypervolume* package (Blonder, 2015) to delineate hypervolumes with two different approaches; (1) using a bandwidth of 0.5 enclosing all occurrences in environmental space (termed *bdw* herein) for delimitation of the multidimensional kernel) or (2) using a multivariate minimum convex polytope (*mcp*). The bandwidth approach assumes that all environmental conditions within the volume enclosed by a multidimensional buffer of 0.5 PC units around the species records is suitable. Alternatively, the *mcp* approach assumes that all conditions within a volume enclosed by the polytope are suitable. All computations were performed using the *raster*,* dismo,* and *hypervolume* packages (Blonder, 2015; Hijmans, 2015; Hijmans, Phillips, Leathwick, & Elith, 2015) for (R Core Team, 2015).

In order to identify those environmental conditions of the native range which are also realized in invasive ranges, and vice versa, we projected the hypervolumes computed for the native and invasive ranges crosswise into geographic space. Assuming some degree of niche conservatism these areas are the most likely areas of origin. At the same time, our approach allows to track any shifts in realized climate niches and to quantify their direction and magnitude.

The predictive performance of the hypervolume models was assessed using three different indices: the area under the receiver operating characteristic curve (AUC; Swets, 1988) ranging from 0.5 (not better than random) to 1.0 (perfect discrimination), the point‐biserial correlation coefficient (COR; Elith et al., 2006) ranging from 0 (no correlation) to 1 (perfect correlation), and Cohen′s Kappa (Allouche, Tsoar, & Kadmon, 2006) ranging from 0 (no agreement) to 1 (perfect). These test statistics were computed for the native and introduced populations using a set of 1,000 random points situated in a circular buffer of 200 km enclosing the occurrence records using the *dismo* package (Hijmans et al., 2015) for Cran R.

## 3 Results

The PCA revealed four PCs with eigenvalues >1 (Table 1) accounting for 90.2% of the total variation. The first PC explained 38.4% of the total variance and was mainly correlated with the maximum temperature of the warmest month and quarter, annual temperature range, annual precipitation, as well as precipitation of the wettest month and quarter. PC 2 (24.1%) was mainly correlated with precipitation of the driest month and quarter, precipitation seasonality and isothermality, whereas PC 3 (17.5%) was mainly correlated with the minimum temperature of the coldest month and quarter. The fourth PC summarizes 10.3% of the total variance and was mainly correlated with the mean temperature of the wettest quarter and precipitation seasonality. For further details, see Table 1.

**Figure 1:**
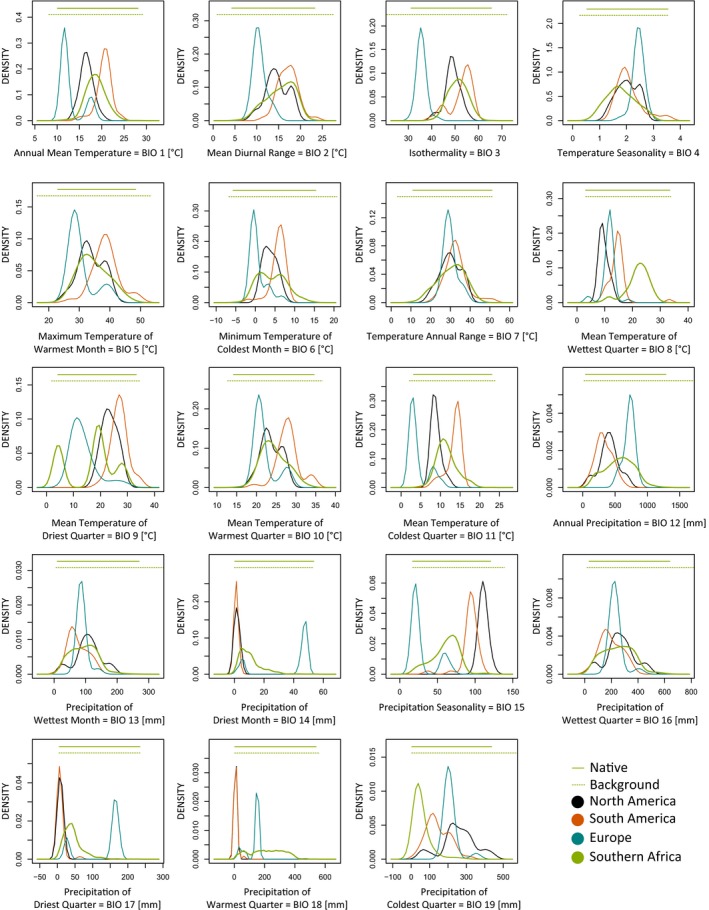
Density profiles for all environmental variables. Background conditions were extracted within a 200‐km buffer enclosing the native species records.

**Figure 2:**
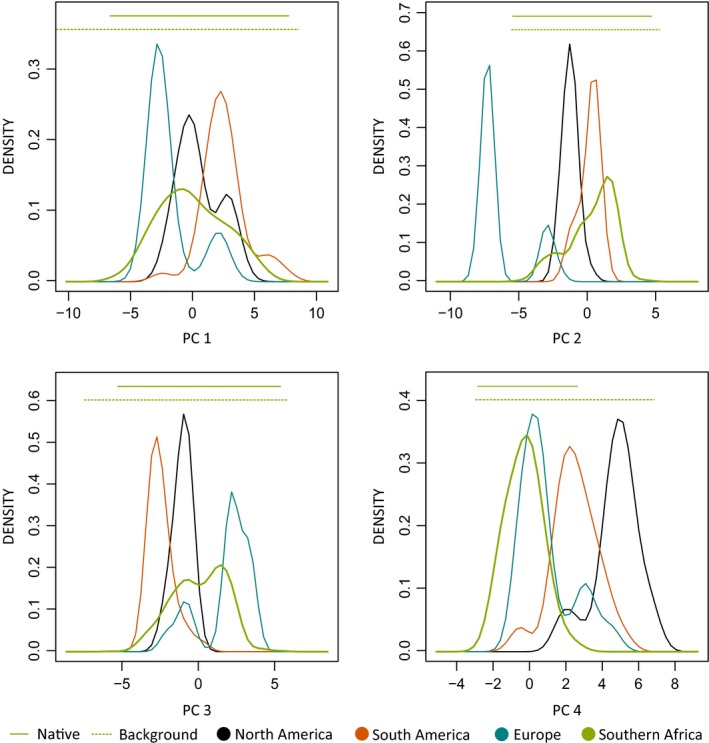
Density plots for the four principal components with Eigenvalues >1. Background conditions were extracted within a 200‐km buffer enclosing the native species records. See Table 1 for details

Univariate comparisons among bioclimatic conditions at native and invaded ranges revealed that the invasive populations are well nested in the variable range of the native population in the mean annual temperature range, temperature seasonality, the maximum temperature of the warmest month, the minimum temperature of the coldest month, the temperature annual range, the mean temperature of the warmest quarter, the annual precipitation, and the precipitation of the wettest month and quarter. However, bioclimatic conditions in at least one invaded region exceed those in the native range in the annual mean temperature, isothermality, mean temperature of the wettest, driest, warmest and coldest quarter, precipitation of the driest month and quarter, precipitation seasonality, and precipitation of the warmest and coldest quarter (Figure 1). The most deviant invasive population were those from Europe. Bimodal peaks in some density plots suggested that European populations can be split into two distinct bioclimatic groups (Figure 1). While the left peak in temperature related variables (BIO 1, 5, 6, 10, 11, and 15, Figure 1) corresponded to the invasive populations from France and Great Britain which are both characterized by oceanic climate, the right peak referred to invasive populations from Portugal and Sicily, areas characterized by a Mediterranean hot climate (Rubel & Kottek, 2010). In precipitation related variables (BIO 14, 17, and 18), this trend was reversed. In environmental space, these patterns were reflected in PC 1 and PC 3 where bioclimatic conditions of invasive populations were nested within those observed in the native range (Figure 2). In PC 2 and PC 4, however, conditions exceeded those of the native range. While in PC 2, the European populations deviated widest from the environmental conditions of the native populations, the North American populations deviated strongest in PC 4.

**Figure 3:**
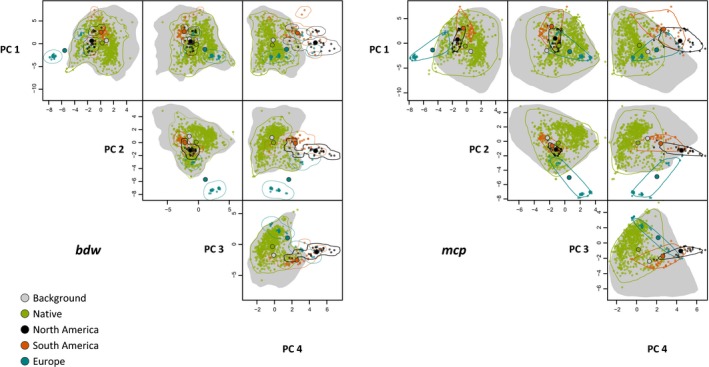
Four dimensional hypervolumes of the environmental niches of *Xenopus laevis* as well as for the potential niche within its native distribution characterizing its available climate space

For European populations, the left peak in the density plot for PC 1 corresponded to Portugal and France while the right corresponded to Sicily. Regarding the second and fourth PCs the first peak of the European populations referred to France while the second related to Portugal and Sicily. The first peak of the European populations of the third PC referred to Portugal and Sicily, while the second corresponded to France (Figure 2). A comparison of the four dimensional hypervolumes of the native and all invaded ranges also revealed the European populations to form two clusters (Figure 3) one of which deviated strongly from the native range.

Test statistics revealed the predictive performance of the four‐dimensional hypervolume models to differ among regions, wherein those computed using the *bdw* approach performed generally better (total range: AUC_bdw_ = 0.827, COR_bdw_ = 0.685, Kappa_bdw_ = 0.640) than those derived from the *mcp* approach (AUC_mcp_ = 0.760, COR_mcp_ = 0.550, Kappa_mcp_ = 0.507). AUC_bdw_ scores for the models computed for the native and the three invasive populations, respectively, ranged between 0.764 and 0.980, while COR_bdw_ values ranged between 0.454 and 0.655, and Kappa_bdw_ between 0.341 and 0.613 for the *mcp* approach, and between 0.672 and 0.847 for AUC_mcp_, from 0.266 to 0.615 for COR_mcp_, and between 0.257 and 0.610 for Kappa_mcp_ (Table 2).

**Table 2:** Statistical tests for the two different hypervolume approaches using the area under the receiver operating characteristic curve (AUC), point‐biserial correlation coefficients (COR), and Cohen′s Kappa

Area	AUC	COR	Kappa
Bandwidth approach (*bdw*)			
Total range	0.827	0.685	0.640
Native	0.764	0.574	0.499
Europe	0.948	0.454	0.341
North America	0.974	0.500	0.400
South America	0.980	0.665	0.613
Multivariate minimum convex polytope approach (*mcp*)			
Total range	0.760	0.550	0.507
Native	0.706	0.454	0.389
Europe	0.686	0.398	0.397
North America	0.672	0.266	0.257
South America	0.847	0.615	0.610

The total volume of the realized niche based on the complete occurrence data set was 395.34 for the *bdw* and 1403.70 for the *mcp* approach. Assessing populations from the native and the three invaded regions separately, models revealed that the native population possesses the largest realized niche volume, followed by the invasive populations in South America. The two approaches revealed different results for Europe and North America (Table 3). Intersections between hypervolumes were largest between the native and the invasive populations from North America, and between populations from North and South America. In the other comparisons intersections were low, yielding low niche overlaps in terms of Soerensen indices (Table 3). The largest centroid distance was observed between populations from North America and Europe and the smallest between populations from North and South America (Table 4). PC 1 had the highest influence on total niche volumes in all cases wherein the contributions of the remaining PCs varied between 0.49 and 0.92 (Table 5).

**Table 3:** Total realized niche volume (displayed in bold); volume of intersecting areas (overlap of environmental/PC space) (below the diagonal); similarity assessed using the Soerensen index (ranging from 0: entirely different to 1: identical; displayed in italics)

	Native	South America	Europe	North America	Background
Bandwidth approach (*bdw*)					
Native	**349.534**	*0.00*	*0.00*	*0.00*	*0.47*
South America	0.17	**22.013**	*0.01*	*0.10*	*0.01*
Europe	0.00	0.13	**11.374**	*0.00*	*0.00*
North America	1.01	1.77	0.00	**14.145**	*0.00*
Background	345.60	0.33	0.22	0.33	**1109.16**
Multivariate minimum convex polytope approach (*mcp*)					
Native	**867.607**	*0.00*	*0.00*	*0.01*	*0.59*
South America	0.00	**10.325**	*0.00*	*0.16*	*0.01*
Europe	0.09	0.00	**3.839**	*0.00*	*0.00*
North America	3.90	0.97	0.00	**2.158**	*0.00*
Background	1270.10	11.47	0.90	0.30	**3014.534**

**Table 4:** Centroid distances (the closer the more similar) for the *mcp* approach (under the diagonal) and the *bdw* approach (above the diagonal)

	Native	South America	Europe	North America
Native		4.85	5.75	4.24
South America	4.17		5.59	2.93
Europe	4.85	4.94		7.18
North America	3.71	2.43	6.14	

**Table 5:** Relative contributions of the PCs to the total realized niche volume

Area	PC 1	PC 2	PC 3	PC 4
Bandwidth approach (*bdw*)				
Total range	1.00	0.67	0.65	0.54
Native	1.00	0.65	0.65	0.49
South America	1.00	0.79	0.76	0.89
Europe	1.00	0.84	0.92	0.87
North America	1.00	0.74	0.74	0.84
Multivariate minimum convex polytope approach (*mcp*)				
Total range	1.00	0.67	0.65	0.54
Native	1.00	0.65	0.65	0.49
South America	1.00	0.79	0.76	0.89
Europe	1.00	0.84	0.92	0.86
North America	1.00	0.73	0.74	0.84

**Figure 4:**
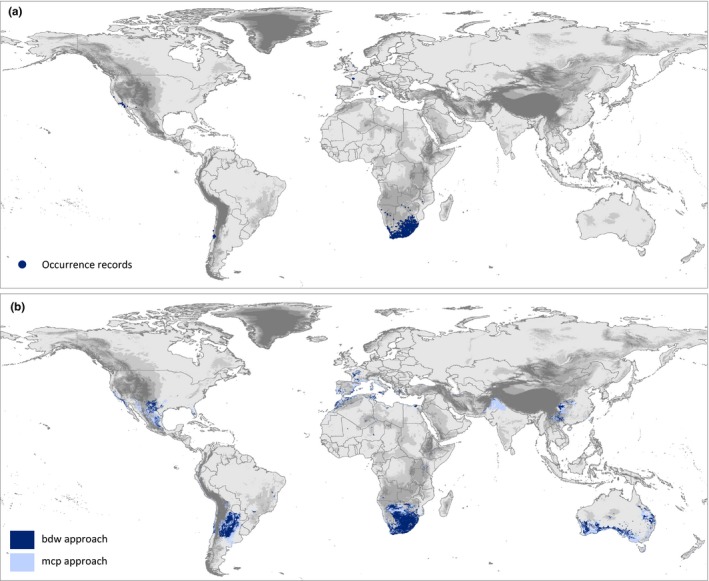
Current global distribution (a) and potential distribution of *Xenopus laevis* as derived from the global hypervolume model trained with the complete set of species records (b)

**Figure 5:**
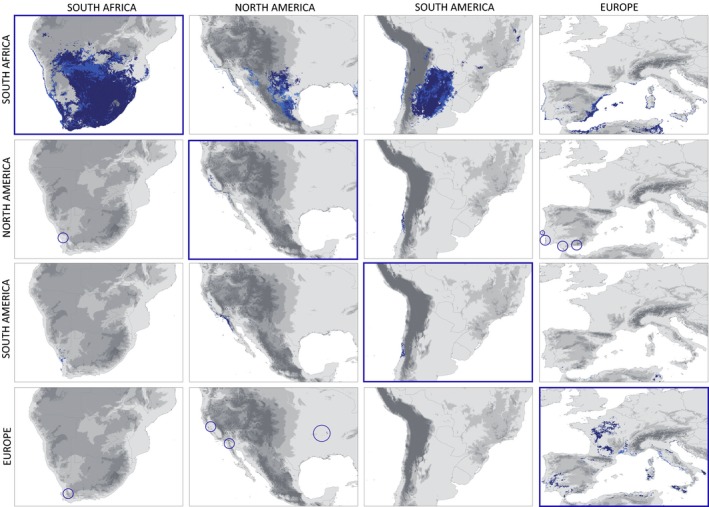
Crosswise projection of *bdw* models (dark blue) and *mcp* models (light blue) for native and invasive populations. The training area is marked by a blue frame and the respective projection areas can be found within the same row. Blue circles indicate the presence of small areas which are within the hypervolume computed for the training range

Using the comprehensive set of pooled native and invasive population records, the hypervolume models predicted not only those areas where the invasive populations are already established but also highlighted additional regions which are potentially suitable for invasion (Figure 4). Areas suggested by both approaches comprise major parts of western Texas and adjacent parts of Mexico, northern parts of Argentina and southern parts of Paraguay, northern Africa and parts of southern coastal Spain, southern and central parts of China, as well as the southern and western coast of Australia. Only the *mcp* approach suggested an additional area in northern India and adjacent Pakistan as suitable for the establishment of invasive populations of *X. laevis* (Figure 4). Training the hypervolume models separately with occurrences from the native or any of the three invaded regions revealed overall similar patterns in the training areas (Figure 5). However, additional suitable areas in Texas and adjacent Mexico, in northern Argentina and southern Paraguay, as well as in northern Africa and along the southern coast of Spain were exclusively predicted by models trained with occurrences from the native range. The results obtained from both the *bdw* and *mcp* approaches revealed overall similar results. Training the models with invasive populations from North America, South America, or Europe separately suggested a potential distribution within the species known distribution range, located near the south‐western Cape region in South Africa (Figure 5). However, crosswise projections also highlighted very different potential distribution sizes depending on the training area, which are congruent with the sizes of the different niche volumes (Table 3).

## 4 Discussion

Our results suggest some degree of realized niche shift between the native and all invaded regions, which is restricted to 10 of 19 bioclimatic variables and well visible in the density plots showing PC 2 and PC 4 (Figure 2). This differentiation is also reflected in the results of the niche overlap analyses. Crosswise projection of the hypervolume models trained in invaded ranges onto the native range revealed the south‐western Cape region as the likely area of origin for all invasive populations.

### 4.1 Niche shifts

Physiological performance of ectothermic taxa, such as amphibians strongly depends on environmental conditions (Feder & Burggren, 1992). Affecting physiological functions such as digestive and locomotive performance, temperature is among the most important extrinsic factors (Angilletta, Niewiarowski, & Navas, 2002; Bennet, 1990; Huey & Kingsolver, 1989). In *X. laevis* and other pipid frogs, temperature was found to affect traits related to fitness like sprint performance (Miller, 1982), swimming velocity (Herrel & Bonneaud, 2012; Wilson et al., 2000), endurance capacity (Herrel & Bonneaud, 2012), reproduction, and larval development rates (Balinsky, 1969).

Previous research suggested *X. laevis* to prefer an ambient temperature of 22 °C (Miller, 1982). Thermal tolerances were found to range from 14 °C to 32 °C (Casterlin & Reynolds, 1980). However, other authors suggested that *X. laevis* withstands more extreme thermal conditions (Nelson et al., 1982), and critical thermal limits of 2 °C and 39 °C, respectively, were obtained for the invasive population in France (J. Courant and A. Herrel, pers. comm.). Laboratory studies indicate that life stages might not be equally affected by extreme thermal conditions (Balinsky, 1969; Wu & Gerhart, 1991) and ontogenetic shifts during embryonic development seem to alter thermal tolerances (Nelson et al., 1982). However, the variability of the critical thermal limits among the native populations of *X. laevis* is not known and available information is limited to adult specimens originating from populations from the Western Cape.

A comparison of the experimentally established preferred temperature range and critical limits with our results reveals the annual mean temperatures in colonized areas to be lower than the species’ thermal preferences except for Chile. However, ambient temperatures generally remain within the critical temperature range of the species. During the warmest quarter temperatures correspond to the species’ preferences in the native range and North America, while remaining lower in Europe and exceeding this range in South America (Figure 1). Maximum monthly temperatures are within the preferred range in most populations but occasionally exceed the species’ critical thermal maximum in South America. However, microhabitats have been found to strongly deviate from conditions of the wider ecosystem, effectively buffering extreme conditions and therefore reducing mortality during extreme weather events (Scheffers, Edwards, Diesmos, Williams, & Evans, 2014), representing a potential for effective short‐time buffering of extremes. On larger time scales such as monthly averages, this buffering capacity may be reduced as the averages of microhabitat temperature and ambient temperature may reach equilibrium. *Xenopus laevis* is predominantly aquatic and the microclimate within the aquatic environment buffers short‐term extremes of air temperatures (Lampert & Sommer, 2007). In lentic habitats, water temperatures in the hypolimnion do not fall below 4 °C (Lampert & Sommer, 2007), effectively shielding inhabitant frogs from lethal air temperatures. Individuals were also reported to actively avoid exposure to critical conditions, for example, by moving into cooler parts of the water body when exposed to high temperatures (47 °C) in Arizona (G. J. Measey, unpublished data) or excavate pits into the soft bottom mud where water temperatures remain lower (McCoid & Fritts, 1980). Albeit freezing may be a major mortality factor (Eggert & Fouquet, 2006), frogs were recorded moving at 5 °C (Wilson et al., 2000) and throughout winter in France (J. Courant and A. Herrel, unpublished data). While adults may still be active and foraging, even under ice‐bound water, development of tadpoles under these conditions is hampered (G. J. Measey, unpublished data). However, the alleged extinction of two invasive populations that persisted for decades in Great Britain was linked to extreme weather events (cold and drought) of successive winters suggesting that climatic conditions exceeded the buffering capacity of the microhabitat inhabited by *X. laevis* (Tinsley, Stott, Viney, Mable, & Tinsley, 2015). The observed deviation in minimum annual temperature between the native and invaded ranges suggests niche shifts of the realized niches approximating the critical minimum temperatures for *X. laevis*.

Induction of reproductive activity is correlated with both temperature and precipitation. Balinsky (1969) suggested that spawning in the native range in Gauteng at approximately 1,000 m a.s.l. can be triggered by a period of rainfall, whereas Green (2002) suggested that ambient temperatures, probably above 20 °C, initiate reproduction in California. However, in the Cape region, ambient temperatures during reproduction remain lower and reproduction is restricted to the winter months. Comparing precipitation patterns of the native and invasive populations, we detected more pronounced niche shifts than when comparing temperatures (five of eight precipitation related variables (62.5%) vs. five of 11 temperature related variables (45.5%). The most pronounced niche shifts were detected in precipitation seasonality (Figure 1), which was considerably lower in Europe (France and Great Britain) but exceeded the native range in North America. In contrast, precipitation of the wettest month was well within the conditions of the native range in all colonized ranges. Generally, a higher total amount of precipitation combined with low precipitation seasonality may increase the permanence of water bodies and therefore promote the establishment of invasive populations.

Reproductive activity differs within the native and invaded ranges. Within the native range, availability of permanent water bodies was limited in the past but recently increased due to the construction of man‐made dams and irrigation canals. Within the invasive range, most populations inhabit artificial permanent water bodies and hence availability of water is frequently not limiting reproductive activity. Temperature represents another potential constraint for reproduction. With water temperatures regularly reaching 20 °C, climatic conditions in California are less extreme than in the species’ native range (McCoid & Fritts, 1980) leading to an almost year‐round reproductive activity with continuous ovulation (McCoid & Fritts, 1989). It was suggested that low ambient temperatures might hamper reproduction (Nelson et al., 1982). However, the species was found to reproduce during most of the year in France with a strong peak during the summer months (April to June; Courant et al., in press). Hence, low winter temperatures may reduce recruitment but do not necessarily prevent the establishment of invasive populations.

### 4.2 Origin of invasive populations

**Figure 6:**
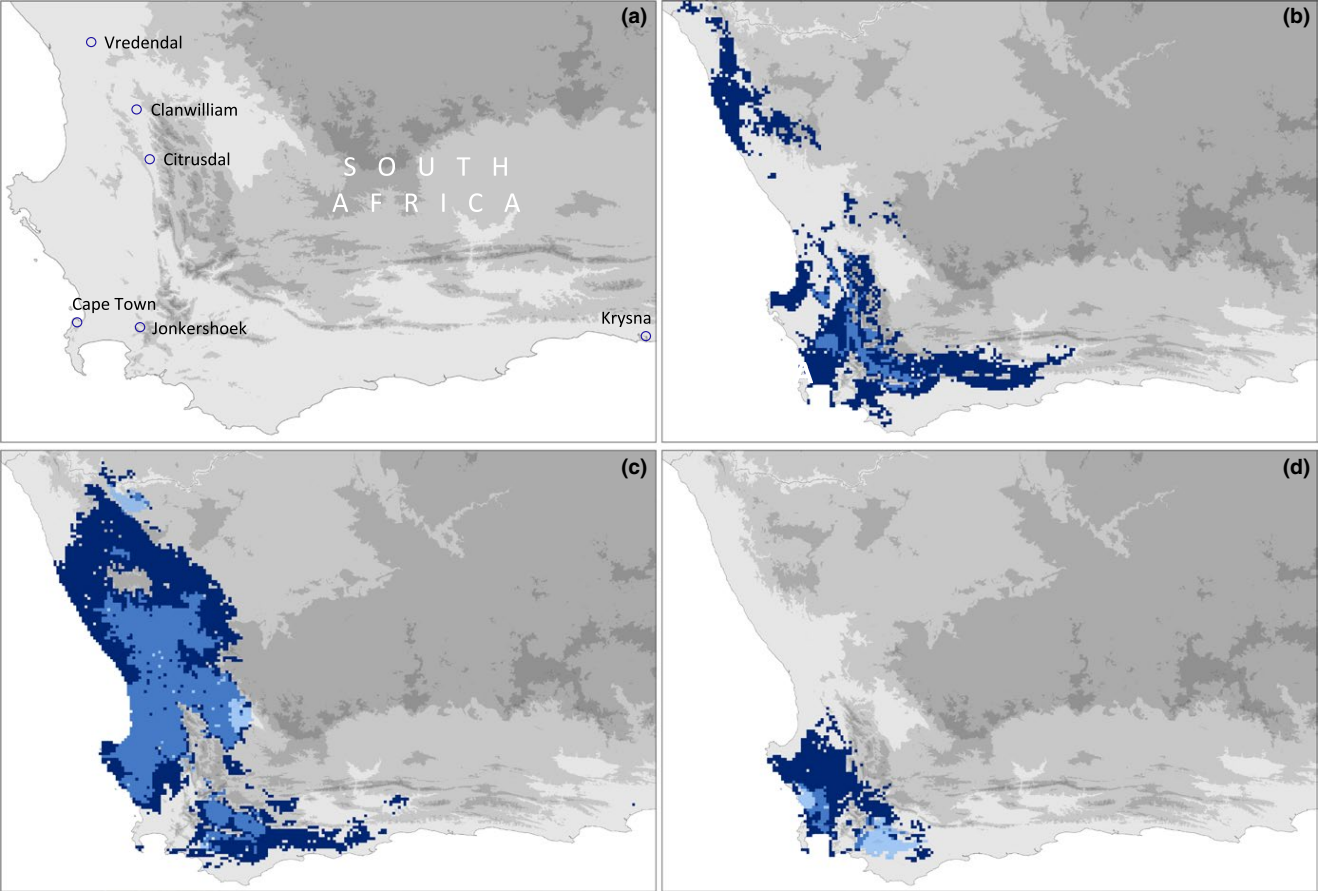
Potential areas of origin of the invasive populations of *Xenopus laevis* across South Africa (a), as well as predicted ranges based on training regions of North America (b), South America (c), and Europe (d).

Environmental conditions may strongly vary across geographic space potentially creating different selective landscapes likely leading to local adaptation and niche differentiation. Therefore, knowledge of the origin of invasive populations may allow an examination if the invaded ranges exhibit similar environmental conditions as the source area. Crosswise projections of the hypervolume models for the invasive populations reveal that parts of the species’ native range resemble environmental conditions found in the invasive ranges. These parts are situated north of Cape Town, in the northern parts of the Western Cape when trained with North or South American populations and close to Clanwilliam/Citrusdal, which is just about 50–100 km southward of Vredendal, when trained with European invasive populations (Figures 4 and 6). This may be the most likely area of origin of the invasive populations according to climate matching when assuming niche conservatism.

As most specimens were exported by a single organization which collected animals within 150 km of Jonkershoek (van Sittert & Measey, 2016), invasive populations were assumed to represent a clade that is distributed between Vredendal in the north to Knysna in the east (Figure 6, Furman et al., 2015). While this clade was already identified in Chile, France, Italy, and Portugal (De Busschere et al., 2016; Lillo et al., 2013; Lobos et al., 2014), the invasive populations in France were recently found to contain another clade from the northern regions of South Africa (De Busschere et al., 2016).

Combining macroecological and molecular evidence facilitates the development of hypotheses about the evolution of the observed niche shifts:

1. Nonadaptive process exposition on historic climate cycles may allow a pre‐adaptation beyond the current realized niche of a species leading to the possibility of acclimatization or phenotypic plasticity, as was suggested for, for example, the invasive freshwater crayfish *Procambarus clarkii* (Chucholl, 2011). From experimental studies, Wilson et al. (2000) observed significant acclimation effects on swimming velocity, suggesting a pre‐adaptation capacity to cooler environments, which may partly explain the invasion success of *X. laevis* in Europe. However, SDM projections of a broad range of different South African amphibian species on paleoclimate scenarios revealed only marginal shifts in potential distributions under last glacial maximum conditions (Mokhatla, Rödder, & Measey, 2015; Schreiner, Rödder, & Measey, 2013) suggesting an overall limited probability of a historic pre‐adaption to cooler environments.
2. A first mechanism involving evolutionary changes would be an establishment of the initial invasive populations in climates similar to the environmental conditions within the area of origin and subsequent adaptation and niche expansion. This scenario is supported by the finding that the invasive populations in, for example, Chile originated from a single introduction and show a signature of a recent bottleneck and are genetically very similar indicating a rapid spread from a small source population (Lobos et al., 2014). Although, multiple introductions occurred in the other invaded areas, in most cases all individuals originated from a single source region. Based on simulations, adaptation to novel environments may be facilitated in initially small population sizes (Holt, Barfield, & Gomulkiewicz, 2005). Selection for a certain beneficial allele might lead to faster fixation in a small population than in a large population. However, before their escape or release, frogs have been kept for several generations under artificial conditions in laboratories or captive breeding facilities, which might also influence the predisposition of the individuals.
3. A second mechanism for rapid adaptation increasing thermal tolerances was suggested by Krehenwinkel et al. (2015). These authors showed that in wasp spiders *Argiope bruennichi* a fundamental niche shift toward significantly lower thermal minima was initiated by a genomic admixture event between two long‐time isolated lineages. This niche shift has facilitated a rapid northward range expansion. A similar phenomenon could explain the invasion success of *X. laevis* in France, where phylogenetic signatures of two different clades were detected (De Busschere et al., 2016). However, experimental data and further genomic analyses are needed to test the hypothesis of rapid adaptation initiated by genomic admixture between long‐time isolated clades.

## 5 Conclusion

In the present study, we demonstrate that invasive populations of *X. laevis* are established well beyond the species’ multivariate realized niche in southern Africa. This finding has important implications for both macroecological niche theory and practical aspects of risk assessments using climate matching.

By suggesting that climate niches remain stable during the invasion process, the observed dynamics in the realized niches of *X. laevis* challenge previous findings. Although we cannot disentangle shifts in the fundamental niche from shifts in the realized niche alone, comparisons with natural history data including environmental tolerances and triggers for reproduction suggest that most niche shifts observed can be explained by realized niche shifts. One exception where this explanation is not evident is the invasive population in France. Here, further studies are required to test whether hybridization of different lineages has enabled a shift in the species’ fundamental niche.

Given the magnitude of the detected niche shifts, the usefulness of climate matching approaches to assess invasion risk is challenged, as it might frequently underestimate the true potential distribution of a species when a geographic subset of the species’ realized distribution is used for model training. As suggested by Broennimann and Guisan (2008) inclusion of all available distribution information may improve predictions but underestimations are still possible. Furthermore, as previous authors have noted a careful analysis of the available environmental space within the training area of the models and a quantification of areas requiring extrapolation beyond this training range may improve the reliability of assessments by exclusion of those areas with high uncertainty (Elith, Kearney, & Phillips, 2010; Measey et al., 2012). Unfortunately, this procedure will restrict the application of climate matching to areas characterized by environmental conditions most similar to the training range and for the majority of other areas predictions which require extrapolation remains unreliable.

From a management point of view, it is possible to assess the reliability of correlative SDMs by comparing the species’ realized environmental niche with what is known from its fundamental niche in terms of physiological performance and physiological limits. As demonstrated herein for *X. laevis*, it can be expected that its true invasion potential is larger than its estimated potential distribution based on its currently realized niche. In this case, the climate matching approach is well able to identify areas with high risk of further invasion but is less reliable in identifying actually unsuitable habitats. Further conservation measures need to prevent additional introductions in suitable habitats which may arise due to anthropogenic climate change (Ihlow et al., 2016), not only within the already invaded regions but also in those areas which are highlighted by our global hypervolume model.

## Conflict of Interest

None declared.

## References

[ece33010-bib-0001] Alexander, J. M. , & Edwards, P. J. (2010). Limits to the niche and range margins of alien species. Oikos, 119, 1377–1386.

[ece33010-bib-0002] Allouche, O. , Tsoar, A. , & Kadmon, R. (2006). Assessing the accuracy of species distribution models: Prevalence, kappa and the true skill statistic (TSS). Journal of Applied Ecology, 43, 1223–1232.

[ece33010-bib-0003] Amaral, P. , & Rebelo, R. (2012). Diet of invasive clawed frog *Xenopus laevis* at Lage stream (Oeiras, W Portugal). Herpetological Journal, 22, 187–190.

[ece33010-bib-0004] Angilletta, M. J. , Niewiarowski, P. H. , & Navas, C. A. (2002). The evolution of thermal physiology in ectotherms. Journal of Thermal Biology, 27, 249–268.

[ece33010-bib-0005] Balinsky, B. I. (1969). The reproductive ecology of amphibians of the Transvaal highveld. Zoologica Africana, 4, 37–93.

[ece33010-bib-0006] Beaumont, L. J. , Hughes, L. , & Poulsen, M. (2005). Predicting species distributions: Use of climatic parameters in BIOCLIM and its impact on predictions of species’ current and future distributions. Ecological Modelling, 186, 250–269.

[ece33010-bib-0007] Bennet, A. F. (1990). Thermal dependence of locomotor capacity. American Journal of Physiology, 259, R253–R258. 2201218 10.1152/ajpregu.1990.259.2.R253

[ece33010-bib-0008] Blonder, B. (2015). Hypervolume: High‐dimensional kernel density estimation and geometry operations. R package version 1.4.1. Retrieved from http://CRAN.R-project.org/package=hypervolume

[ece33010-bib-0009] Blonder, B. (2016). Do hypervolumes have holes? The American Naturalist, 187, E93–E105. 10.1086/685444 27028084

[ece33010-bib-0010] Blonder, B. , Lamanna, C. , Violle, C. , & Enquist, B. J. (2014). The n‐dimensional hypervolume. Global Ecology and Biogeography, 23, 595–609.

[ece33010-bib-0011] Bomford, M. , Barry, S. C. , & Lawrence, E. (2010). Predicting establishment success for introduced freshwater fishes: A role for climate matching. Biological Invasions, 12, 2559–2571.

[ece33010-bib-0012] Bowman, A. W. , & Azzalini, A. (2014). R package ‘sm’: Nonparametric smoothing methods. R packege version 2.2‐5.4. Retrieved from http://www.stats.gla.ac.uk/~adrian/sm, http://azzalini.stat.unipd.it/Book_sm

[ece33010-bib-0013] Broennimann, O. , & Guisan, A. (2008). Predicting current and future biological invasions: Both native and invaded ranges matter. Biology Letters, 4, 585–589. 18664415 10.1098/rsbl.2008.0254 PMC2610080

[ece33010-bib-0014] Broennimann, O. , Treier, U. A. , Müller‐Schärer, H. , Thuiller, W. , Peterson, A. T. , & Guisan, A. (2007). Evidence of climatic nich shift during biological invasion. Ecology Letters, 10, 701–709. 17594425 10.1111/j.1461-0248.2007.01060.x

[ece33010-bib-0015] Busby, J. R. (1991). BIOCLIM – a bioclimatic analysis and prediction system In Margules C. R., & Austin M. P. (Eds.), Nature conservation: Cost effective biological surveys and data analysis (pp. 64–68). CSIRO: Melbourne.

[ece33010-bib-0017] Casterlin, M. E. , & Reynolds, W. W. (1980). Diel activity and thermoregulatory behavior of a fully aquatic frog – *Xenopus laevis* . Hydrobiologia, 75, 189–191.

[ece33010-bib-0018] Chucholl, C. (2011). Population ecology of an alien “warm water” crayfish (*Procambarus clarkii*) in a new cold habitat. Knowledge and Management of Aquatic Ecosystems, 401, 29.

[ece33010-bib-0019] Colwell, R. , & Rangel, T. (2009). Hutchinson's duality: The once and future niche. Proceedings of the National Academy of Sciences, USA, 106, 19651–19658. 10.1073/pnas.0901650106 PMC2780946 19805163

[ece33010-bib-0501] Courant, J. , Secondi, J. , Bezeiriat, V. , & Herrel, A. (in press). Resources allocated to reproduction decrease at the range edge of an expanding population of an invasive amphibian. Biological Journal of the Linnean Society.

[ece33010-bib-0020] Crisp, M. D. , & Cook, L. G. (2012). Phylogenetic niche conservatism: What are the underlying evolutionary and ecological causes? New Phytologist, 196, 681–694. 22943495 10.1111/j.1469-8137.2012.04298.x

[ece33010-bib-0021] De Busschere, C. , Courant, J. , Herrel, A. , Rebelo, R. , Rödder, D. , Measey, G. J. , & Backeljau, T. (2016). Unequal contribution of native South African phylogeographic lineages to the invasion of the African clawed frog, *Xenopus laevis*, in Europe. PeerJ, 4, e1659. 26855879 10.7717/peerj.1659 PMC4741087

[ece33010-bib-0022] Deblauwe, V. , Droissart, V. , Bose, R. , Sonké, B. , Blach‐Overgaard, A. , Svenning, J.‐C. , … Couvreur, T. L. P. (2016). Remotely sensed temperature and precipitation data improves species distribution modelling in the tropics. Global Ecology and Biogeography, 25, 443–454.

[ece33010-bib-0023] Early, R. , & Sax, D. F. (2014). Climatic niche shifts between species’ native and naturalized ranges raise concern for ecological forecasts during invasions and climate change. Global Ecology and Biogeography, 23, 1356–1365.

[ece33010-bib-0024] Eggert, C. , & Fouquet, A. (2006). A preliminary biotelemetric study of a feral invasive *Xenopus laevis* population in France. Alytes, 23, 144–149.

[ece33010-bib-0025] Elith, J. , Graham, C. H. , Anderson, R. P. , Dudik, M. , Ferrier, S. , Guisan, A. , Hijmans, R. J. , et al. (2006). Novel methods improve prediction of species′ distributions form occurrence data. Ecography, 29, 129–151.

[ece33010-bib-0026] Elith, J. , Kearney, M. , & Phillips, S. (2010). The art of modelling range‐shifting species. Methods in Ecology and Evolution, 1, 330–342.

[ece33010-bib-0027] Feder, M. E. , & Burggren, W. W. (1992). Environmental physiology of the amphibians. Chicago: University of Chicago Press.

[ece33010-bib-0500] Fisher, M. C. , & Garner, T. W. (2007). The relationship between the emergence of Batrachochytrium dendrobatidis, the international trade in amphibians and introduced amphibian species. Fungal Biology Reviews, 21(1), 2–9.

[ece33010-bib-0028] Fitzpatrick, M. C. , Weltzin, J. F. , Sanders, N. J. , & Dunn, R. R. (2007). The biogeography of prediction error: Why does the introduced range of the fire ant over‐predict its native range? Global Ecology and Biogeography, 16(1), 24–33.

[ece33010-bib-0029] Fouquet, A. , & Measey, G. J. (2006). Plotting the course of an African clawed frog invasion in Western France. Animal Biology, 56, 95–102.

[ece33010-bib-0030] Furman, B. L. S. , Bewick, A. J. , Harrison, T. L. , Greenbaum, E. , Gvozdik, V. , Kusamba, C. , & Evans, B. J. (2015). Pan‐African phylogeography of a model organism, the African clawed frog *‘Xenopus laevis’* . Molecular Ecology, 24, 909–925. 25583226 10.1111/mec.13076

[ece33010-bib-0031] Green, S. L. (2002). Factors affecting oogenesis in the South African Clawed frog (*Xenopus laevis*). Comparative Medicine, 52, 307–312. 12211272

[ece33010-bib-0033] Guisan, A. , Petitpierre, B. , Broennimann, O. , Daehler, C. , & Kueffer, C. (2014). Unifying niche shift studies: Insights from biological invasions. Trends in Ecology and Evolution, 29, 260–269. 24656621 10.1016/j.tree.2014.02.009

[ece33010-bib-0034] Herrel, A. , & Bonneaud, C. (2012). Temperature dependence of locomotor performance in the tropical clawed frog, *Xenopus tropicalis* . Journal of Experimental Biology, 215, 2465–2470. 22723486 10.1242/jeb.069765

[ece33010-bib-0035] Hijmans, R. J. (2015). Raster: Geographic data analysis and modeling. R package version 2.5‐2. Retrieved from http://CRAN.R-project.org/package=raster

[ece33010-bib-0036] Hijmans, R. J. , Phillips, S. , Leathwick, J. , & Elith, J. (2015). Dismo: Species distribution modeling. R package version 1.0‐12. Retrieved from http://CRAN.R-project.org/package=dismo

[ece33010-bib-0037] Holt, R. D. , Barfield, M. , & Gomulkiewicz, R. (2005). Theories of niche conservatism and evolution: Could exotic species be potential tests? In Sax D., Stachowicz J. & Gaines S. D. (Eds.), Species invasions: Insight into ecology, evolution, and biogeography (pp. 259–290). Sunderland, MA: Sinauer Associates.

[ece33010-bib-0038] Huey, R. B. , & Kingsolver, J. G. (1989). Evolution of thermal sensitivity of ectotherm performance. Trends in Ecology and Evolution, 4, 131–135. 21227334 10.1016/0169-5347(89)90211-5

[ece33010-bib-0039] Hutchinson, G. E. (1957). Concluding remarks. Cold Spring Harbor Symposia on Quantitative Biology, 22, 415–427.

[ece33010-bib-0040] Ihlow, F. , Courant, J. , Secondi, J. , Herrel, A. , Rebelo, R. , Measey, G. J. , … Rödder, D. (2016). Impacts of climate change on the global invasion potential of the African clawed frog *Xenopus laevis* . PLoS One, 11(6), e0154869. 27248830 10.1371/journal.pone.0154869 PMC4889038

[ece33010-bib-0041] Jimenez‐Valverde, A. , Peterson, A. T. , Soberon, J. , Overton, J. M. , Aragon, P. , & Lobo, J. M. (2011). Use of niche models in invasive species risk assessments. Biological Invasions, 13, 2785–2797.

[ece33010-bib-0043] Kopecký, O. , Patoka, J. , & Kalous, L. (2016). Establishment risk and potential invasiveness of the selected exotic amphibians from pet trade in the European Union. Journal of Nature Conservation, 31, 22–28.

[ece33010-bib-0044] Kozak, K. H. , Graham, C. H. , & Wiens, J. J. (2008). Integrating GIS‐based environmental data into evolutionary biology. Trends in Ecology and Evolution, 23, 141–148. 18291557 10.1016/j.tree.2008.02.001

[ece33010-bib-0045] Krehenwinkel, H. , Rödder, D. , & Tautz, D. (2015). Eco‐genomic analysis of the poleward range expansion of the wasp spider *Argiope bruennichi* shows rapid adaptation and genomic admixture. Global Change Biology, 21, 4320–4332. 26183328 10.1111/gcb.13042

[ece33010-bib-0046] Kumschick, S. , & Richardson, D. M. (2013). Species‐based risk assessments for biological invasions: Advances and challenges. Diversity and Distributions, 19, 1095–1105.

[ece33010-bib-0047] Lampert, W. , & Sommer, U. (2007). Limnoecology – The ecology of lakes and streams. Oxford: Oxford University Press.

[ece33010-bib-0048] Lavergne, S. , & Molofsky, J. (2007). Increased genetic variation and evolutionary potential drive the success of an invasive grass. Proceedings of the National Academy of Science, USA, 104, 3883–3888. 10.1073/pnas.0607324104 PMC1805698 17360447

[ece33010-bib-0502] Lillo, F. , Faraone, F. P. , & Valvo, M. L. (2011). Can the introduction of Xenopus laevis affect native amphibian populations? Reduction of reproductive occurrence in presence of the invasive species. Biological Invasions, 13(7), 1533–1541.

[ece33010-bib-0049] Lillo, F. , Dufresnes, C. , Faraone, F. P. , Lo Valvo, M. , & Stock, M. (2013). Identification and potential origin of invasive clawed frogs *Xenopus* (Anura: Pipidae) in Sicily based on mitochondrial and nuclear DNA. Italian Journal of Zoology, 80, 566–573.

[ece33010-bib-0050] Lobos, G. , Mendez, M. A. , Cattan, P. , & Jaksic, F. (2014). Low genetic diversity of the successful invasive African clawed frog *Xenopus laevis* (Pipidae) in Chile. Studies on Neotropical Fauna and Environment, 49, 50–60.

[ece33010-bib-0051] McCoid, M. J. , & Fritts, T. H. (1980). Observations of feral populations of *Xenopus laevis* (Pipidae) in southern California. Bulletin of the Southern California Academy of Sciences, 79, 82–86.

[ece33010-bib-0052] McCoid, M. J. , & Fritts, T. H. (1989). Growth and fat‐body cycles in feral populations of the african clawed frog, *Xenopus laevis* (Pipidae), in California with comments on reproduction. Southwestern Naturalist, 34, 499–505.

[ece33010-bib-0200] Measey, J. (2016). Overland movement in African clawed frogs (Xenopus laevis): a systematic systematic review. PeerJ, 4:e2474 27688972 10.7717/peerj.2474 PMC5036101

[ece33010-bib-0053] Measey, G. J. , Rödder, D. , Green, S. L. , Kobayashi, R. , Lillo, F. , Lobos, G. , … Thirion, J. M. (2012). Ongoing invasions of the African clawed frog, *Xenopus laevis*: A global review. Biological Invasions, 14, 2255–2270.

[ece33010-bib-0054] Measey, G. J. , & Tinsley, R. C. (1998). Feral *Xenopus laevis* in south Wales. Herpetological Journal, 8, 23–27.

[ece33010-bib-0055] Measey, G. J. , Vimercati, G. , De Villiers, F. A. , Mokhatla, M. M. , Davies, S. J. , Edwards, S. , & Altwegg, R. (2015). Frog eat frog: Exploring variables influencing anurophagy. PeerJ, 3, e1204. 26336644 10.7717/peerj.1204 PMC4556157

[ece33010-bib-0056] Measey, G. J. , Vimercati, G. , De Villiers, F. A. , Mokhatla, M. , Davies, S. J. , Thorp, C. J. , … Kumschick, S. (2016). A global assessment of alien amphibian impacts in a formal framework. Diversity and Distributions, 22, 970–981.

[ece33010-bib-0057] Medley, K. A. (2010). Niche shifts during the global invasion of the Asian tiger mosquito, *Aedes albopictus* Skuse (Culicidae), revealed by reciprocal distribution models. Global Ecology and Biogeography, 19, 122–133.

[ece33010-bib-0058] Miller, K. (1982). Effect of temperature on sprint performance in the frog *Xenopus laevis* and the Salamander *Necturus maculosus* . Copeia, 1982, 695–698.

[ece33010-bib-0059] Mitchell, C. E. , Agrawal, A. A. , Bever, J. D. , Gilbert, G. S. , Hufbauer, R. A. , Klironomos, J. N. , et al. (2006). Biotic interactions and plant invasions. Ecology Letters, 9, 726–740. 16706916 10.1111/j.1461-0248.2006.00908.x

[ece33010-bib-0060] Mokhatla, M. M. , Rödder, D. , & Measey, G. J. (2015). Assessing the effects of climate change on distributions of Cape Floristic Region amphibians. South African Journal of Science, 111, 171–177.

[ece33010-bib-0061] Nelson, L. , Mild, K. H. , & Lovtrup, S. (1982). Changes in temperature tolerance during the development of *Xenopus laevis* embryos. Journal of Experimental Zoology, 222, 103–104.

[ece33010-bib-0062] Pearman, P. B. , Guisan, A. , Broennimann, O. , & Randin, C. F. (2008). Niche dynamics in space and time. Trends in Ecology and Evolution, 23, 149–158. 18289716 10.1016/j.tree.2007.11.005

[ece33010-bib-0063] Peterson, A. T. (2011). Ecological niche conservatism: A time‐structured review of evidence. Journal of Biogeography, 38, 817–827.

[ece33010-bib-0064] Peterson, A. T. , Papes, M. , & Soberón, J. (2015). Mechanistic and correlative models of ecological niches. European Journal of Ecology, 1, 28–38.

[ece33010-bib-0065] Petitpierre, B. , Kueffer, C. , Broennimann, O. , Randin, C. , Daehler, C. , & Guisan, A. (2012). Climatic niche shifts are rare among terrestrial plant invaders. Science, 335, 1344–1348. 22422981 10.1126/science.1215933

[ece33010-bib-0066] R Core Team (2015). R: A language and environment for statistical computing. Vienna, Austria: R Foundation for Statistical Computing https://www.R-project.org/

[ece33010-bib-0068] Rödder, D. , & Lötters, S. (2009). Niche shift versus niche conservatism? Climatic characteristics of the native and invasive ranges of the Mediterranean house gecko (*Hemidactylus turcicus*). Global Ecology and Biogeography, 18, 674–687.

[ece33010-bib-0069] Rödder, D. , & Lötters, S. (2010). Explanative power of variables used in species distribution modelling: An issue of general model transferability or niche shift in the invasive Greenhouse frog (*Eleutherodactylus planirostris*). Naturwissenschaften, 97, 781–796. 20617298 10.1007/s00114-010-0694-7

[ece33010-bib-0070] Rödder, D. , Schmidtlein, S. , Veith, M. , & Lötters, S. (2009). Alien invasive slider turtle in unpredicted habitat: A matter of niche shift or of predictors studied? PLoS One, 4, e7843. 19956684 10.1371/journal.pone.0007843 PMC2776975

[ece33010-bib-0071] Rödder, D. , Solé, M. , & Böhme, W. (2008). Predicting the potential distribution of two alien invasive Housegeckos (Gekkonidae: *Hemidactylus frenatus*,* Hemidactylus mabouia*). North‐Western Journal of Zoology, 4, 236–246.

[ece33010-bib-0072] Rubel, F. , & Kottek, M. (2010). Observed and projected climate shifts 1901–2100 depicted by world maps of the Köppen‐Geiger climate classification. Meteorologische Zeitschrift, 19, 135–141.

[ece33010-bib-0073] Scheffers, B. R. , Edwards, D. P. , Diesmos, A. , Williams, S. E. , & Evans, T. A. (2014). Microhabitats reduce animal's exposure to climate extremes. Global Change Biology, 20, 495–503. 24132984 10.1111/gcb.12439

[ece33010-bib-0074] Schreiner, C. , Rödder, D. , & Measey, G. J. (2013). Using modern models to test Poynton's predictions. African Journal of Herpetology, 62, 49–62.

[ece33010-bib-0075] Schulte, U. , Hochkirch, A. , Lötters, S. , Rödder, D. , Schweiger, S. , Weimann, T. , & Veith, M. (2012). Cryptic niche conservatism among evolutionary lineages of an invasive lizard. Global Ecology and Biogeography, 21, 198–211.

[ece33010-bib-0076] Sillero, N. , Reis, M. , Vieira, C. P. , Vieira, J. , & Morales‐Hojas, R. (2014). Niche evolution and thermal adaptation in the temperate species *Drosophila americana* . Journal of Evolutionary Biology, 27, 1549–1561. 24835376 10.1111/jeb.12400

[ece33010-bib-0077] van Sittert, L. , & Measey, G. J. (2016). Historical perspectives on global exports and research on African clawed frogs (*Xenopus laevis*). Transactions of the Royal Society of South Africa, 71, 157–166.

[ece33010-bib-0078] Soberón, J. , & Nakamura, M. (2009). Niches and distributional areas: Concepts, methods, and assumptions. Proceedings of the National Academy of Sciences, USA, 106, 19644–19650. 10.1073/pnas.0901637106 PMC2780935 19805041

[ece33010-bib-0079] Strubbe, D. , Broennimann, O. , Chiron, F. , & Matthysen, E. (2013). Niche conservatism in non‐native birds in Europe: Niche unfilling rather than niche expansion. Global Ecology and Biogeography, 22, 962–970.

[ece33010-bib-0080] Swets, K. (1988). Measuring the accuracy of diagnostic systems. Science, 240, 1285–1293. 3287615 10.1126/science.3287615

[ece33010-bib-0081] Thomas, C. D. , Cameron, A. , Green, R. E. , Bakkenes, M. , Beaumont, L. J. , Collingham, Y. C. , Erasmus, B. F. N. , et al. (2004). Extinction risk from climate change. Nature, 427, 145–148. 14712274 10.1038/nature02121

[ece33010-bib-0082] Tingley, R. , Vallinoto, M. , Sequeira, F. , & Kearney, M. R. (2014). Realized niche shift during a global biological invasion. Proceedings of the National Academy of Sciences, USA, 111, 10233–10238. 10.1073/pnas.1405766111 PMC4104887 24982155

[ece33010-bib-0083] Tinsley, R. C. , Stott, L. C. , Viney, M. E. , Mable, B. K. , & Tinsley, M. C. (2015). Extinction of an introduced warm‐climate alien species, *Xenopus laevis*, by extreme weather events. Biological invasions, 17(11), 3183–3195. 26430383 10.1007/s10530-015-0944-x PMC4581400

[ece33010-bib-0084] Urban, M. , Phillips, B. , Skelly, D. , & Shine, R. (2008). A toad more traveled: The heterogeneous invasion dynamics of cane toads in Australia. The American Naturalist, 171, 134–148. 10.1086/527494 18271722

[ece33010-bib-0085] Vandepitte, K. , De Meyer, T. , Helsen, K. , Van Acker, K. , Roldan‐Ruiz, I. , Mergeay, J. , & Honnay, O. (2014). Rapid genetic adaptation precedes the spread of an exotic plant species. Molecular Ecology, 23, 2157–2164. 24479960 10.1111/mec.12683

[ece33010-bib-0504] Vogt, S. , de Villiers, F. A. , Ihlow, F. , Rödder, D. , & Measey, J. (2017). Competition and feeding ecology in two sympatric Xenopus species (Anura: Pipidae). PeerJ, 5: e3130. https://doi.org/10.7717/peerj.3130 28439453 10.7717/peerj.3130 PMC5399871

[ece33010-bib-0087] Wiens, J. J. , & Graham, C. H. (2005). Niche conservatism: Integrating evolution, ecology, and conservation biology. Annual Review of Ecology, Evolution, and Systematics, 36, 519–539.

[ece33010-bib-0088] Wilson, R. S. , James, R. S. , & Johnston, I. A. (2000). Thermal acclimation of locomotor performance in tadpoles and adults of the aquatic frog *Xenopus laevis* . Journal of Comparative Physiology B‐Biochemical Systemic and Environmental Physiology, 170, 117–124. 10.1007/s003600050266 10791571

[ece33010-bib-0505] Wilson, J. R. U. , Dormontt, E. E. , Prentis, P. J. , Lowe, A. J. , & Richardson, D. M. (2009). Something in the way you move: dispersal pathways affect invasion success. Trends in Ecology & Evolution, 24(3), 136–144. 19178981 10.1016/j.tree.2008.10.007

[ece33010-bib-0089] Wu, M. , & Gerhart, J. (1991). Raising *Xenopus* in the laboratory. Methods in Cell Biology, 36, 3–18. 1811140 10.1016/s0091-679x(08)60269-1

